# Bio-Inspired Dark Adaptive Nighttime Object Detection

**DOI:** 10.3390/biomimetics9030158

**Published:** 2024-03-03

**Authors:** Kuo-Feng Hung, Kang-Ping Lin

**Affiliations:** Electrical Engineering Department, Chung Yuan Christian University, Taoyuan City 320314, Taiwan; terry.hung@cycu.org.tw

**Keywords:** bio-inspired, dark adaptation, object detection

## Abstract

Nighttime object detection is challenging due to dim, uneven lighting. The IIHS research conducted in 2022 shows that pedestrian anti-collision systems are less effective at night. Common solutions utilize costly sensors, such as thermal imaging and LiDAR, aiming for highly accurate detection. Conversely, this study employs a low-cost 2D image approach to address the problem by drawing inspiration from biological dark adaptation mechanisms, simulating functions like pupils and photoreceptor cells. Instead of relying on extensive machine learning with day-to-night image conversions, it focuses on image fusion and gamma correction to train deep neural networks for dark adaptation. This research also involves creating a simulated environment ranging from 0 lux to high brightness, testing the limits of object detection, and offering a high dynamic range testing method. Results indicate that the dark adaptation model developed in this study improves the mean average precision (mAP) by 1.5−6% compared to traditional models. Our model is capable of functioning in both twilight and night, showcasing academic novelty. Future developments could include using virtual light in specific image areas or integrating with smart car lighting to enhance detection accuracy, thereby improving safety for pedestrians and drivers.

## 1. Introduction

The Earth’s day and night cycle necessitates adaptations in living organisms to cope with the changing light conditions for survival. During the daytime, abundant sunlight allows visual organs in creatures to function optimally, enabling them to carry out normal activities. However, nighttime presents a challenge with insufficient light, causing many creatures to perceive dim surroundings, restrict their nocturnal movements, and face potential threats from predators.

Some animals, such as moths, frogs, and geckos, possess night vision capabilities that allow them to distinguish colors even in low-light conditions [1]. Mice, for instance, have larger pupils [2], which enable more light to enter their eyes. Moreover, the mouse retina contains a greater number of rod cells specialized for sensing light intensity rather than color, making mice highly sensitive to low-light environments. This sensitivity is advantageous for hunting and avoiding predators. In contrast, human eyes have fewer rod cells, making them less proficient in low-light conditions compared to ones that dominate during daylight. Consequently, humans often rely on methods like increasing ambient visible light or using infrared night vision glasses [3] to improve nighttime visibility.

When pedestrians are active at night, the lack of illumination makes it challenging for drivers to spot them, particularly in areas without streetlights or in low-light conditions. Additionally, vehicle headlights may create glare, impeding a driver’s ability to see pedestrians or obstacles ahead. According to Jessica Cicchino of the Insurance Institute for Highway Safety (IIHS) in their 2022 report, up to 75% of pedestrian fatalities in car accidents occur at night or in poorly lit environments [4]. In incidents involving nighttime pedestrian injuries, 87% of drivers reported that they could not see the pedestrians at the time of the accident. Furthermore, the pedestrian detection performance of vehicles equipped with Automatic Emergency Braking (AEB) systems is less effective in darkness. The IIHS is in the process of developing the first official nighttime pedestrian collision prevention rating for such environments.

To address the challenges of nighttime detection, a common approach involves combining software and hardware techniques, such as image enhancement, data augmentation, domain transfer, and deep learning methods to improve recognition rates. In terms of light sources, enhancing nighttime object characteristics can be achieved by adding external infrared light sources or combining visible and infrared light. Alternatively, high-sensitivity sensors can be employed to capture clear and high-quality images in low-light environments.

However, the adoption of multiple sensors like thermal imaging, laser radar, and 3D depth cameras can be expensive and challenging to implement in various industries, often incurring additional costs. This raises the question of whether alternative methods, such as emulating the dark adaptation mechanisms found in organisms and early training and learning, could open new avenues for research and development in this field.

The literature review indicates that the extensive research in enhancing low-light imaging has predominantly focused on traditional approaches like software enhancements, hardware improvements, and the integration of different light sources. Common methods include image enhancement through data augmentation, domain transfer, and the utilization of deep learning techniques to elevate recognition rates under nocturnal conditions. Traditional light sources have been supplemented with external infrared and combined visible-infrared lighting to improve object detectability at night. However, these conventional methods often come with high costs and complex implementation challenges, necessitating the search for innovative solutions. Despite the advancements in employing high-sensitivity sensors to capture clearer images in dim conditions, the significant financial and technical barriers remain a concern for widespread application, particularly in safety-critical areas like pedestrian and vehicle detection.

The gap in current methodologies presents an opportunity for bio-inspired solutions, particularly those that emulate the natural dark adaptation processes observed in nocturnal animals. This biological perspective has been underexplored, especially in applying these mechanisms to enhance low-light detection technologies. The literature indicates a few attempts at leveraging biomimicry, such as the development of biomimetic compound eyes for motion detection and the study of nocturnal insects’ visual systems for signal enhancement. However, these studies have not fully translated into practical low-light detection applications. By integrating bio-inspired models with advanced image processing techniques, such as image fusion and gamma correction, this study proposes to bridge the gap between biological efficiency and technological advancement. This approach is poised to open new pathways for the development of night vision systems, providing a cost-effective and energy-efficient alternative to the traditional methods currently in use.

The innovation in this research lies in mimicking the human eye’s mechanism by overlaying the original image captured by the camera (left eye) with an image adjusted for contrast (right eye) to create an enhanced image for object detection. The fused image combines both original and contrast-enhanced features, effectively improving recognition rates. Furthermore, the study emulates the mechanism of dark adaptation and utilizes pre-gamma correction to allow deep neural networks to learn from images before and after dark adaptation. Finally, this research addresses the significant variations in ambient brightness at night by constructing a simulated environment ranging from 0 lux to high brightness, providing a high dynamic range testing method and contributing to academic research.

The global market size of autonomous vehicles exceeded USD 27 billion in 2021 and is expected to continue growing, potentially reaching nearly USD 62 billion by 2026. Night vision recognition technology plays a crucial role in enabling autonomous vehicles to identify roads, pedestrians, animals, and obstacles in low-light or no-light conditions. This technology enhances safety, driving efficiency, and the overall riding experience, helping prevent potential traffic accidents during nighttime driving.

The following sections of this paper will be presented in the following order: Section 2—The Literature Review; Section 3—Methodology; Section 4—Research Results; Section 5—Discussion; and Section 6—Conclusions.

## 2. The Literature Review

A novel biomimetic compound eye system for moving object detection has been proposed [5]. This section discusses the design scheme, the development of biomimetic control circuits, and the mathematical model of the biomimetic compound eye. This model uses multiple ommatidia (sub-eyes) to capture several targets simultaneously and utilizes repetitive imaging to reduce random errors. For night vision capabilities, this article does not specifically mention the use of nighttime imagery.

Ref. [6] of this article delves into how nocturnal insects maintain efficient visual functions in extremely low-light environments, highlighting their unique adaptations in optical design, neural adaptations, and signal processing within their visual systems. To enhance the reliability of visual signals, nocturnal insects may integrate signals over space and time at higher levels of their visual systems.

This article focuses on pedestrian detection using visible and far-infrared (FIR) cameras under different lighting conditions, both during the day and at night [7]. This research reveals that infrared images play a crucial role in improving accuracy. When using a Support Vector Machine (SVM) detector with only visible light images from the CVC-14 dataset, the error rate is remarkably high at 71.8%. However, when infrared images are incorporated, the error rate significantly drops to 25.4%.

The Yolo-v5 algorithm, based on deep learning, is employed for pedestrian detection in both day and night scenarios [8]. It comprises two sub-networks, one for processing RGB color images and the other for handling IR thermal images. The information from these sub-networks is combined into a multispectral approach, resulting in a more accurate pedestrian detector. According to this study, the mean average precision (mAP) indicator shows a significant improvement, with a score of 53.3% using the KAIST dataset compared to using RGB images alone.

Winston Chen and colleagues discussed object detection technology in low-light environments [9]. Their research suggests that basic image enhancement techniques, like histogram equalization, have little impact on detection results and can even reduce the mAP accuracy from 29% to 27.8%. The reason is that such enhancements are not adaptive. While adaptive methods like CycleGAN show slight improvements, they require extensive training.

Another article explores object detection in low-light environments, focusing on the performance of the YOLOv3 algorithm [10]. This study utilizes the Exclusively Dark (ExDark) dataset for testing and compares the results with the COCO dataset. Various image enhancement techniques are explored, including histogram equalization, dynamic histogram equalization, and exposure fusion framework. The mAP value of YOLOv3 on the ExDark dataset is 21.35%, which is significantly lower than the 57.9% achieved on the COCO dataset. Even though histogram equalization may appear more noticeable to the naked eye, it reduces the mAP of YOLOv3 to only 19.69%, but using multiple exposure fusion slightly improves it to 20.49%.

Kefu Yi et al. use data enhancement to enhance low-light images at night and bridge the gap in nighttime vehicle/pedestrian detection data [11]. They employ domain transfer techniques to reduce the differences between daytime and nighttime data, improving the mAP. However, these methods require substantial data preparation and processing time, and their effectiveness in adapting to the changing nighttime environment still needs verification.

Martin Solar et al. have patented an auxiliary light source for a vehicle vision system [12]. This system includes a camera installed on the vehicle’s windshield, an Electronic Control Unit (ECU) with an image processor, and an auxiliary light source. The ECU processes camera data, identifies lane markings, and detects objects ahead of the vehicle. If an object is in the vehicle’s path, the auxiliary light source is activated to enhance visibility, thereby improving object detection accuracy.

In the industrial era, high-sensitivity sensors have been employed to enhance object identification in low-light conditions. SONY STARVIS, a CMOS sensor technology [13] designed for low-light environments, including nighttime, stands out for its performance and ability to capture high-quality images. STARVIS 2, an advanced version, offers an even wider dynamic range.

High Dynamic Range (HDR) is a technique used in imaging and photography to reproduce a greater dynamic range of luminosity than is possible with standard digital imaging or photographic techniques [14]. It has been pivotal in enhancing visibility under diverse lighting conditions, particularly beneficial in nighttime object detection scenarios. HDR techniques typically involve capturing and combining multiple photographs with varying exposure levels to produce a single image that represents a broader range of luminance levels than standard digital imaging techniques. However, traditional HDR methods can be limited by their need for multiple exposures and high computational costs, making them less viable for real-time applications. In contrast, our approach aims to simulate the HDR effect by adopting bio-inspired mechanisms mimicking human night vision. This novel methodology enables the enhancement of single-exposure images, reducing the need for multiple captures and, thus, aligning more closely with the dynamic requirements of real-time nighttime object detection.

In conclusion, achieving nighttime object recognition involves utilizing image enhancement, data augmentation, domain transfer, and other technologies, along with deep learning methods, to enhance recognition rates. In the realm of light sources, improvements can be made by adding external infrared light sources, combining visible and infrared light, or using high-sensitivity sensors to capture clear and high-quality images in low-light conditions. While these traditional methods often incur additional costs, there is potential to explore alternative approaches, such as emulating the dark adaptation mechanisms found in organisms and early training and learning, which are the primary focus of this article.

## 3. Methodology

### 3.1. Biological Dark Adaptation and Binocular Vision

**Figure 1:**
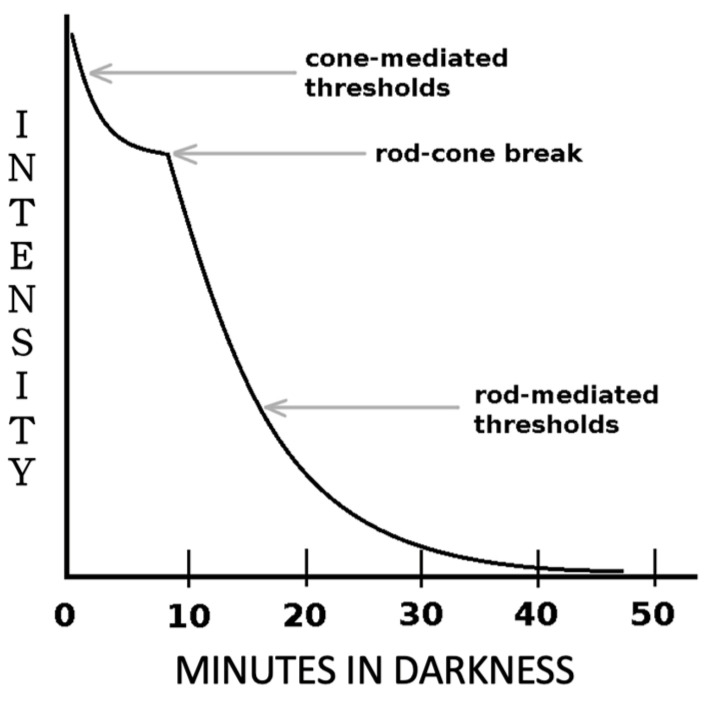
Visual Response to Darkness.

Dark adaptation is a biological mechanism that allows our eyes to adjust to changes in ambient light, particularly in low-light environments [15] (see Figure 1). When we enter a dark area, our body initiates a series of physiological processes to enhance our ability to see, including the following mechanisms:

1. Pupil dilation: The pupil, the circular opening in the iris, regulates the amount of light entering the eye. In a dark environment, the pupil dilates rapidly to allow more light in, increasing the amount of light received by the photosensitive cells on the retina. This enhances brightness and contrast, making it easier to distinguish objects;
2. Photosensitivity adjustment: Dark adaptation also involves adjustments in color perception. In low light, our visual system favors rod cells, which are sensitive to brightness but not color. This causes objects in dark spaces to appear more gray;
3. Viewpoint movement: In a dark environment, you might notice small changes in your viewpoint because retinal neurons become more sensitive to light changes. This adaptation helps us stay alert in low-light situations and aids in capturing potential threats or important information.

**Figure 2:**
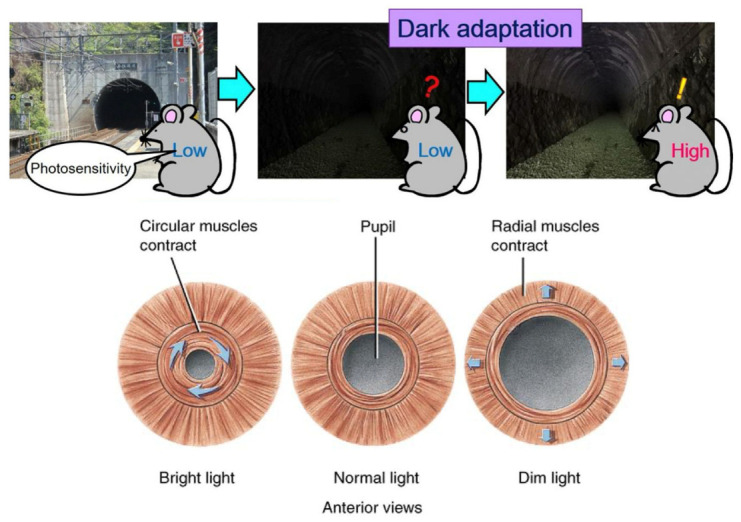
Dark adaptation and pupil changes.

These mechanisms work together to ensure our ability to see and identify objects in the dark, as shown in Figure 2, improving our chances of survival and adaptability to our surroundings.

**Figure 3:**
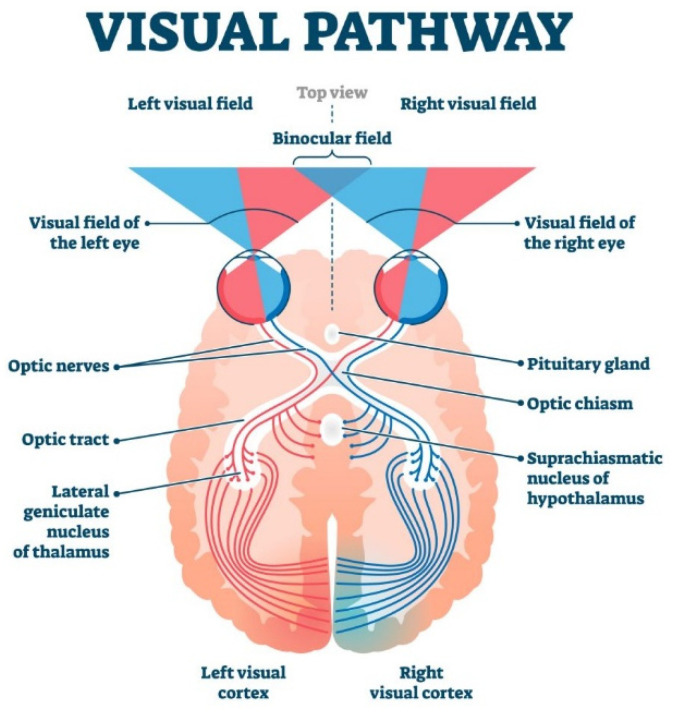
Binocular vision.

Binocular vision, on the other hand, refers to the collaboration of both eyes to merge the images they capture into a single, clear image [16]. This not only provides a wider field of view but also enhances depth perception, as presented in Figure 3. Neurons in both eyes respond more strongly when they perceive similar basic visual features, such as shape, edges, color, or motion.

In the realm of contrast sensitivity [17], a study compared the performance of one eye versus both eyes under varying stimulus contrasts, evaluating the extent of binocular advantage. The results showed that binocular dominance increased as contrast decreased, particularly with a significant interaction effect on fixation duration in the lowest contrast conditions. Recent research has suggested that binocular vision offers benefits not only for depth perception and vision [18] but also for cognition and language. This has led to suggestions that the advantages of binocular vision should be applied in educational and rehabilitative contexts.

### 3.2. Research Datasets

**Figure 4:**
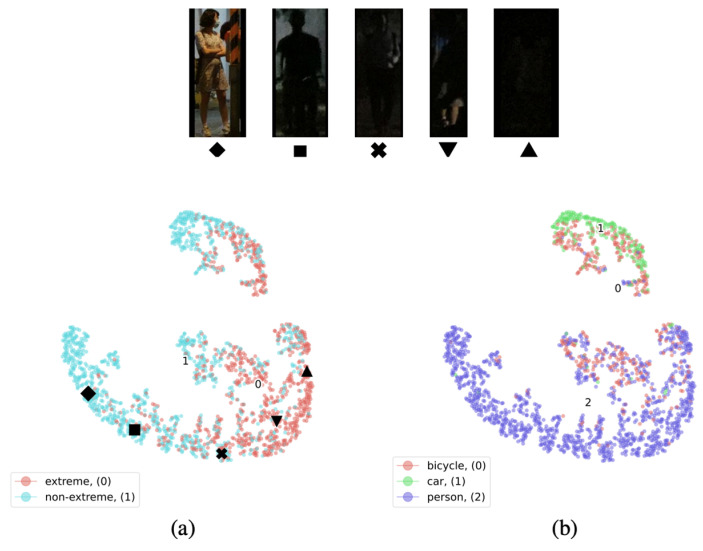
NOD dataset with different low-light conditions and classes.

The Night Object Detection Dataset (NOD database) is a valuable resource designed specifically for object detection in low-light conditions, providing high-quality and extensive data [19]. This dataset comprises over 7000 images categorized into three groups: people; bicycles; and cars. All these photos were taken in the evening on streets, presenting varying degrees of low-light conditions (see Figure 4). The NOD database serves as a crucial resource for researchers working on the development of object detection technologies in extremely low-light scenarios. Its significance extends to enhancing the performance of nighttime surveillance systems, autonomous driving technology, and other applications reliant on low-light visual recognition.

**Figure 5:**
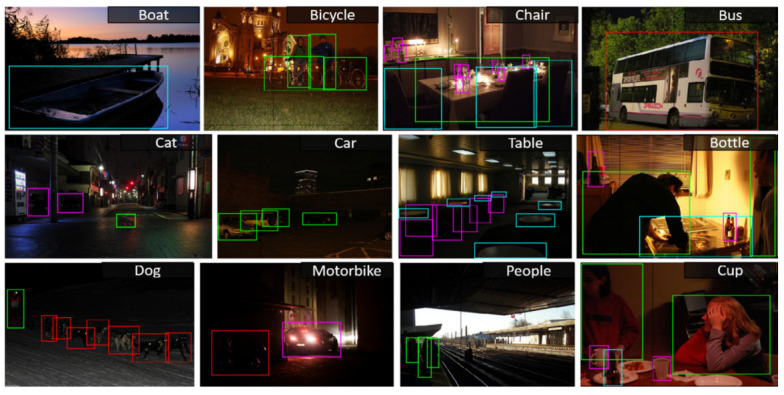
ExDark dataset images and object level annotations.

For image processing testing, researchers often turn to the NOD database. However, when it comes to the study and validation of deep learning methods, the ExDark (Extended Dark) dataset becomes a popular choice [20]. One possible reason is that the ExDark dataset encompasses 12 common categories encountered in nighttime scenarios, offering a broader range of light variations, including twilight and low-light, as presented in Figure 5.

Object detection in low-light environments poses a significant challenge, and the ExDark dataset is tailor-made to address this challenge. This dataset primarily focuses on images captured in twilight, low-light, nighttime, or dark settings. To tackle the complexities of accurate target detection and classification in such environments, the dataset includes 12 categories, such as people, cars, and dogs, covering a wide range of potential applications. It serves as a valuable resource for evaluating and enhancing target detection algorithms in low-light conditions, making it relevant for autonomous driving, surveillance systems, image enhancement, and research purposes. The dataset comprises 3740 training images, 741 validation images, and additional test images for comprehensive evaluation.

### 3.3. Image Processing

Detecting objects in low-light or nighttime conditions presents a considerable challenge, primarily because the quality of images in such environments tends to be lower. This lower image quality can make identifying and tracking objects difficult. In low-light situations, images often exhibit characteristics like high noise, reduced contrast, and limited color information. These factors can significantly impact the accuracy and efficiency of object detection. To address these challenges, researchers have explored various image enhancement techniques to improve nighttime vision.

**Figure 6:**
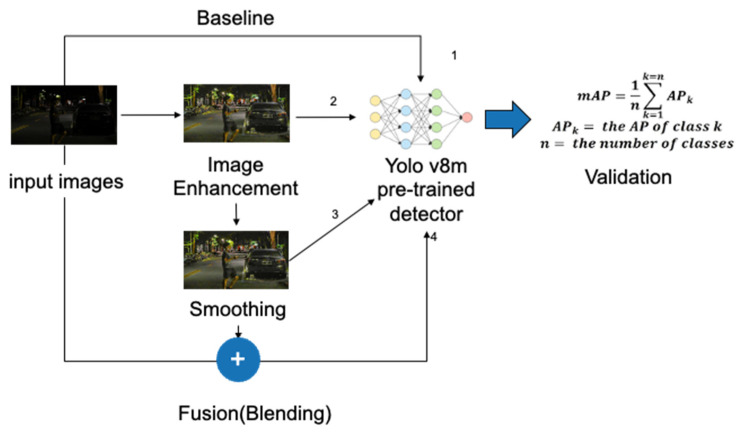
Image processing flowchart.

In this section, we delve into image processing methods, including the comparison of original images, image enhancement, image smoothing, and image overlay fusion, as shown in Figure 6. We utilize the Yolo v8m [21] pre-trained model for object detection. The pre-trained data set used for this purpose is derived from the COCO Dataset [22], a widely recognized resource for training and evaluating deep learning models in object detection, instance segmentation, and key point detection. The COCO Dataset encompasses a wide range of object categories, boasts a substantial number of annotated images, and employs standardized evaluation metrics.

#### 3.3.1. Image Enhancement

**Figure 7:**
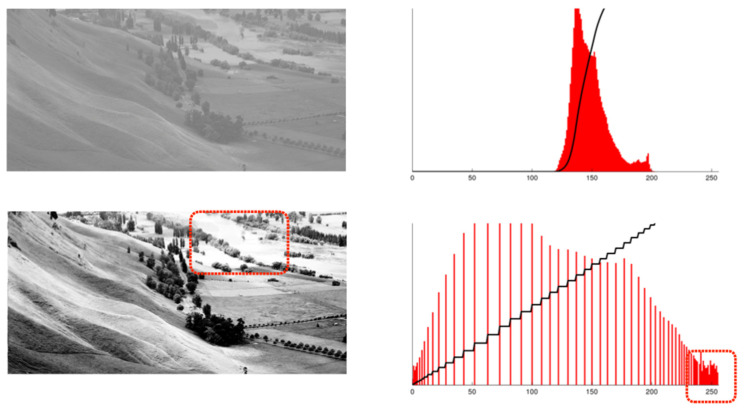
Histogram equalization and over-exposure problem indicated by dotted red box.

Histogram equalization (HE) is a widely used image enhancement technique that enhances an image’s contrast by redistributing its gray values. This technology effectively makes low-contrast areas in an image clearer, thereby improving object visibility and detection, particularly in low-light environments. Figure 7 demonstrates an original image before and after the HE process [23]. The bottom shows over-exposure in the dotted box where the details are missing compared to the original image.

**Figure 8:**
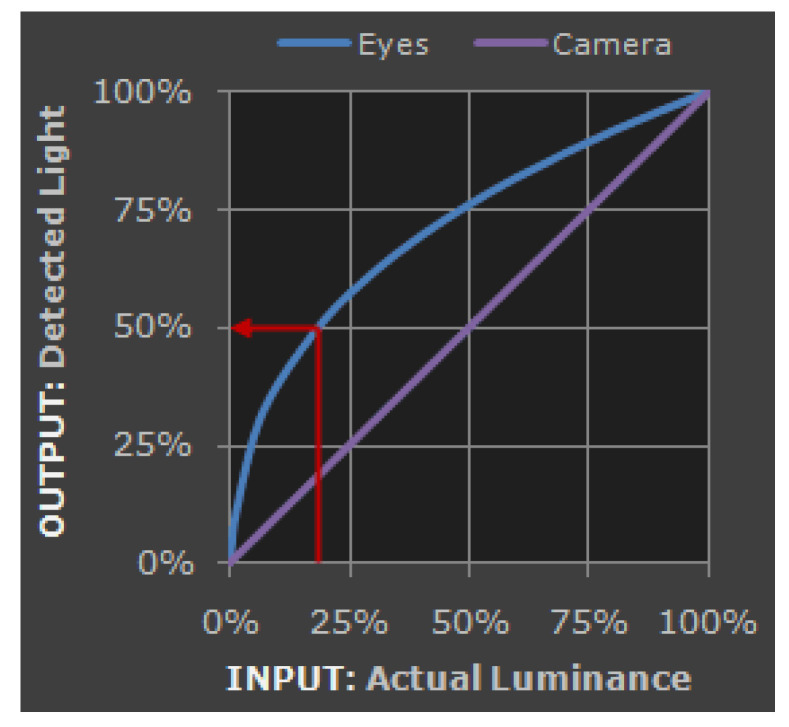
Nonlinear input and output phenomena of Gamma correction.

Gamma correction is another common technique in image processing, primarily used to adjust the brightness and contrast of images to make them appear more natural or aligned with human visual perception, as shown in Figure 8. This correction is based on the nonlinear characteristics of the human visual system. In areas with low brightness, our eyes are much more sensitive to changes in brightness compared to well-lit areas. Gamma correction adjusts the image’s brightness and contrast using an exponential function known as the gamma function, typically represented by the equation below:

$$V_{out}=A\cdot V_{in}^{\gamma }$$

**Figure 9:**
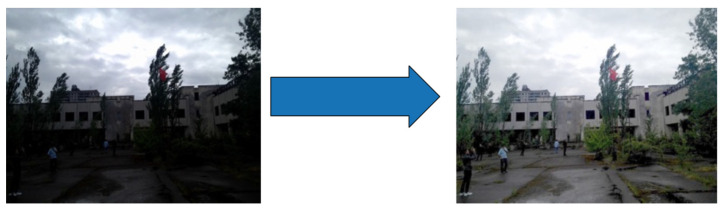
Gamma-correction image processing.

In this equation [24], A is a constant, usually set to 1; $V_{in}$ is the input pixel value; $V_{out}$ is the output pixel value, and γ is the gamma value. Gamma correction enhances image display, making it appear more realistic and in harmony with our visual perception (see Figure 9).

However, it is worth noting that applying histogram equalization or gamma correction to nighttime images can lead to increased noise, overexposure, and other issues. In this study, an alternative image enhancement method called LIME (low-light image enhancement) [25] was used. LIME is primarily designed for enhancing images captured in low-light conditions. It works by determining the maximum value for each pixel in the R, G, and B channels to estimate the individual pixel’s illumination. Then, structural priors are applied to refine the initial illumination map, resulting in the final illumination map.

$$E\left(x\right)=\frac{I\left(x\right)}{\underset{J}{\min}\left(\sum_{x}|J\left(x\right)-\max\left(R\left(x\right),G\left(x\right),B\left(x\right)\right)\left|+\lambda \sum_{xy}w\left(x,y\right)|J\left(x\right)-J\left(y\right)\right|\right)}$$

In this equation,

$E\left(x\right)$ represents the enhanced image;

$I\left(x\right)$ represents the original low-light image;

$J\left(x\right)$ represents the illumination map;

$J\left(y\right)$ represents the refined illumination map;

λ is a regularization parameter;

$w\left(x,y\right)$ is a weight function dependent on the structural similarity between x and y.

#### 3.3.2. Image Smoothing

**Figure 10:**
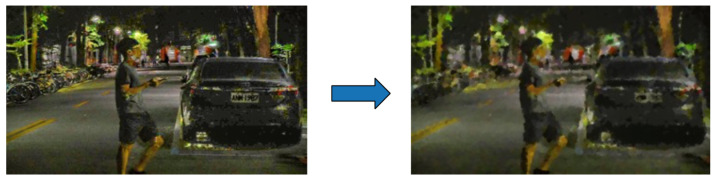
Image smoothing.

After adjusting the brightness or contrast in nighttime images, noise issues often arise. To mitigate these noise problems, image smoothing [26] can be employed to introduce a level of blur by calculating the average value of each pixel along with its neighboring pixels in the image (see Figure 10). This process is achieved through an average blur, which is a linear filter that works by summing the pixel values within a defined area and then dividing by the total number of pixels in that area. This approach effectively reduces noise in the image, resulting in a smoother and cleaner result.

$$B\left(i,j\right)=\frac{1}{k^{2}}\overset{a}{\underset{m=-a}{\sum }}\overset{b}{\underset{n=-b}{\sum }}I\left(i+m,j+n\right)$$

where

- $B\left(i,j\right)$ is the pixel value at position $\left(i,j\right)$ in the blurred image;
- $I\left(i+m,j+n\right)$ is the pixel value at position $\left(i+m,j+n\right)$ in the original image;
- $a=\frac{k-1}{2}$ and $b=\frac{k-1}{2}$ are the radii of the convolution kernel;
- k is the size of the convolution kernel.

In this study, the image that undergoes image enhancement is further processed using smoothing techniques.

#### 3.3.3. Image Fusion

Image fusion is used to mimic the human eye’s mechanism by overlaying the original image captured by the camera (real eye) with an image adjusted for contrast (virtual eye) to create a combined image for object detection. This combination involves a weighted sum, where each image carries a specific weight, determining its significance in the result.

$$\mathrm{dst}\left(x,y\right)=\alpha \cdot \mathrm{src}1\left(x,y\right)+\beta \cdot \mathrm{src}2\left(x,y\right)$$

In this equation

- $\mathrm{dst}\left(x,y\right)$ is the pixel value at position $\left(x,y\right)$ in the output image;
- $\mathrm{src}1\left(x,y\right)$ and $\mathrm{src}2\left(x,y\right)$ are the pixel values at position $\left(x,y\right)$ in the two input images, respectively;
- α and β are the weights for the two images, and their values typically lie between 0 and 1. Usually, α+β=1 to ensure that the pixel values remain within the range of 0 to 255;
- γ is an offset value, which is typically set to 0.

**Figure 11:**
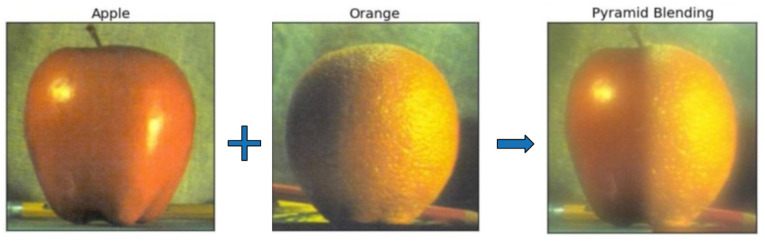
Image Fusion by Pyramid Blending.

An example of Image fusion is shown in Figure 11. Apple and orange can be fused to a new object with the features of apple and orange as well. In this study, we designate the original image as ‘src1’, while the second image, ‘src2’, is derived from the smoothed image.

### 3.4. Deep Learning Model and Process Design

#### 3.4.1. Introduction to YOLO

The YOLO (You Only Look Once) algorithm is an efficient deep learning method for object detection [21]. The ability to quickly identify abnormal activities or specific objects is crucial for ensuring safety during nighttime operations. Traditional object detection methods rely on manually engineered features, while deep learning can automatically learn features from data, which is especially valuable in dynamic and complex nighttime environments. Combining YOLO with other sensor technologies like infrared and thermal imaging further enhances nighttime object detection. Multimodal learning integrates information from various sources, providing a more comprehensive understanding of the nighttime environment. Transfer learning enables a YOLO model pre-trained on a large, labeled dataset to be applied to nighttime object detection tasks, saving training time and leveraging previously acquired knowledge to improve detection accuracy.

To augment nighttime training data, data augmentation techniques can be employed. For instance, simulating different lighting and weather conditions can enhance the generalizability of deep learning models. In this research, various lighting scenarios are designed to allow the model to adapt to changing environments during training. During the validation phase, a dataset spanning from extremely low brightness to high brightness, representing environments with illumination levels below 1 lux, is created. Additionally, a comprehensive virtual testing environment is established, contributing to academic research in the field.

#### 3.4.2. Training, Verification, and Testing Processes

**Figure 12:**
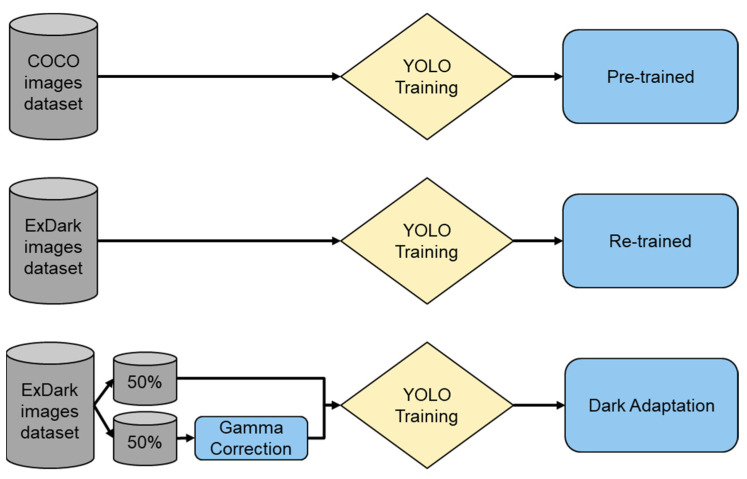
Dataset arrangement for training process.

In this study, three models were utilized: COCO; ExDark; and ExDark with dark adaptation. Given that COCO already provides a pre-trained model, we can use it directly. For the ExDark model training, we used 3740 images with Yolo v8. Furthermore, we divided these 3740 trained images into two groups, with 50% undergoing gamma correction, while the remaining 50% remained unchanged. The resulting model, trained on all 3740 images, was named the ExDark with DA (dark adaptation) (see Figure 12).

**Figure 13:**
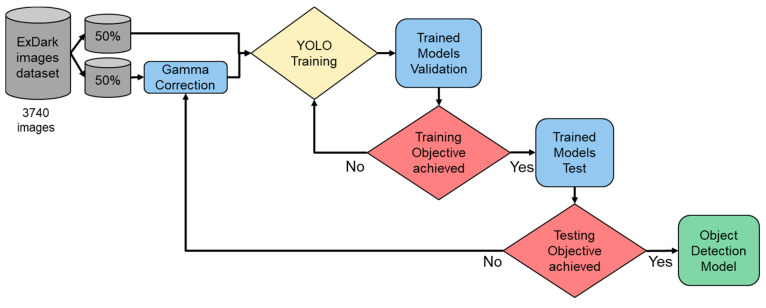
Flowchart of our re-trained dark adaptation model.

For the three aforementioned models, we employed ExDark validation images for verification purposes, comprising a total of 741 images. If the desired performance was not achieved, we fine-tuned parameters such as the learning rate, batch size, number of iterations, and hyperparameters. The overall process is illustrated in Figure 13.

After training the model, we conducted further investigations into its robustness. Nighttime images typically exhibit two characteristics: extremely low brightness, approaching 0 lux; and significant variations in brightness. As there is currently no dedicated test database available, this study devised a testing method to simulate real-world changes in light source intensity. We subjected the test images to a range of lighting conditions, varying from low brightness to high brightness, in order to evaluate the model’s resilience.

The mean average precision (mAP) serves as a critical metric for assessing the accuracy of object detection models, particularly in target detection frameworks like YOLO (You Only Look Once). The mAP takes into account the model’s precision (precision) and recall (recall) across multiple categories, offering a comprehensive performance evaluation by calculating the average precision (AP) for each category and averaging them. Essentially, mAP gauges the model’s capability to detect objects across various categories, considering both its accuracy and rate of misses. A higher mAP indicates superior performance in multi-category object detection tasks, signifying the model’s effectiveness in identifying and locating objects from diverse categories.

In mAP50, a prediction is deemed correct only when the Intersection over Union (IoU) between the predicted bounding box and the actual object box reaches 50% or more. This calculation method is relatively stringent, as it requires a substantial overlap between the predicted frame and the actual object for it to be considered a valid detection. Consequently, this study employs mAP50 as the *mAP* metric.

$$mAP=\frac{1}{n}\overset{k=n}{\underset{k=1}{\sum }}AP_{k}\;$$

$$AP_{k}=the\;AP\;of\;class\;k$$

n=the number of classes

### 3.5. How Dark Is Dark?

Lux is a unit of measurement for light intensity, representing the amount of luminous flux per square meter of surface. Typically, lux is measured using a lux meter or illuminometer, devices capable of sensing light intensity and converting it into lux values. Lux plays a crucial role by providing a standardized way to quantify light intensity. This measurement finds applications in various fields, including lighting design, photography, and plant growth research, helping individuals understand and manage the impact of light in diverse scenarios.

**Figure 14:**
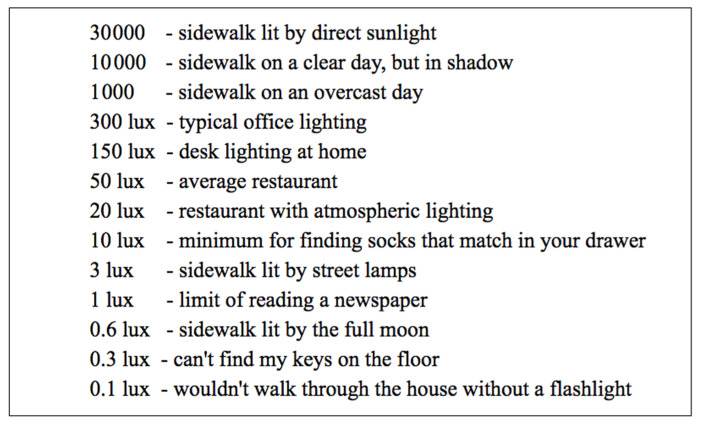
Personnel activity in different lighting (lux) environments.

As per Google research [27], in an environment with 30 lux, capturing a single photo becomes challenging, and even with the use of HDR+ technology, merging multiple photos does not yield satisfactory results in environments below 3 lux. HDR+ is a photography technology used by Google on its Pixel phones. It improves image quality by merging multiple photos taken at different exposure times, enhancing details, especially in shadows, and reducing noise without user interaction. The primary goal of this technology is to enhance photography in conditions ranging from 3 lux to 0.3 lux. It can be inferred that in extremely low-light environments, human visual capabilities are severely restricted, impacting fundamental activities such as photography and navigation (see Figure 14).

### 3.6. High Dynamic Range Simulation and Testing

Indoors at night, where people are active, the lighting is typically around 20 lux, owing to limited illumination. However, the situation is different outdoors, particularly in urban settings, where one encounters large LED screens, stadium floodlights, vehicle headlights, and more. In such outdoor environments, the brightness levels can soar to around 700 lux [28]. This level of brightness surpasses that of a typical office setting during daylight hours and is akin to the brightness observed on cloudy days. Consequently, assessing activities at night necessitates high dynamic range simulations. This is primarily because there is currently no existing test dataset that adequately covers both daytime and nighttime conditions.

$$I_{\mathrm{adjusted}}\left(x,y\right)=f\left(I_{\mathrm{original}}\left(x,y\right),L\left(\theta ,\phi \right),A\left(x,y,\theta ,\phi ,r\right)\right)$$

where

- $I_{\mathrm{adjusted}}\left(x,y\right)$ and $I_{\mathrm{original}}\left(x,y\right)$ are the adjusted and original pixel intensities at a location $\left(x,y\right)$, respectively;
- $L\left(\theta ,\phi \right)$ is the light intensity at angles $\left(\theta \right)\;$and $\left(\phi \right)$;
- $A\left(x,y,\theta ,\phi ,r\right)$ is an angle and position-dependent light attenuation function at angles $\left(\theta \right)$, $\left(\phi \right)$, and $\left(r\right)$ reflectivity;
- $\left(f\right)\;$is a nonlinear function that describes the complex interaction between the light and the image pixels.

**Figure 15:**
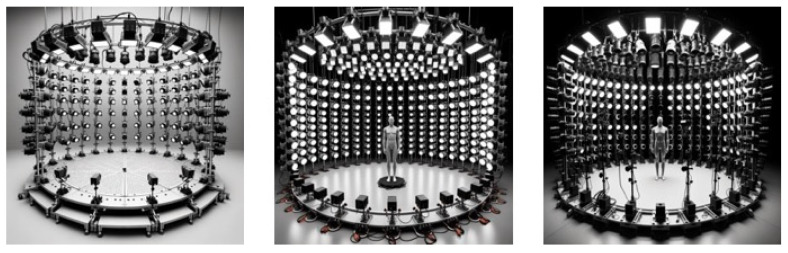
Surrounding intensity simulation.

Figure 15 shows the simulation from different intensities and angles.

In the images within the ExDark dataset, the angle between the object and the light source remains unknown. Consequently, we simplify the aforementioned equation by assuming that the light source illuminates the object in a parallel fashion from infinity. The adjustment of image intensity is then simplified into a linear enhancement and deviation, controlled by parameters α and δ, for achieving image brightness adjustment, as elaborated below.

$$I_{\mathrm{new}}\left(x,y\right)=\alpha \;\cdot \;I_{\mathrm{original}}\left(x,y\right)+\delta$$

where

- $I_{original}\left(x,y\right)$ is the original image pixel intensity;
- $I_{\mathrm{new}}\left(x,y\right)$ is the adjusted image pixel intensity;
- α is the scale factor for controlling image brightness;
- δ is the delta for controlling brightness.

**Figure 16:**
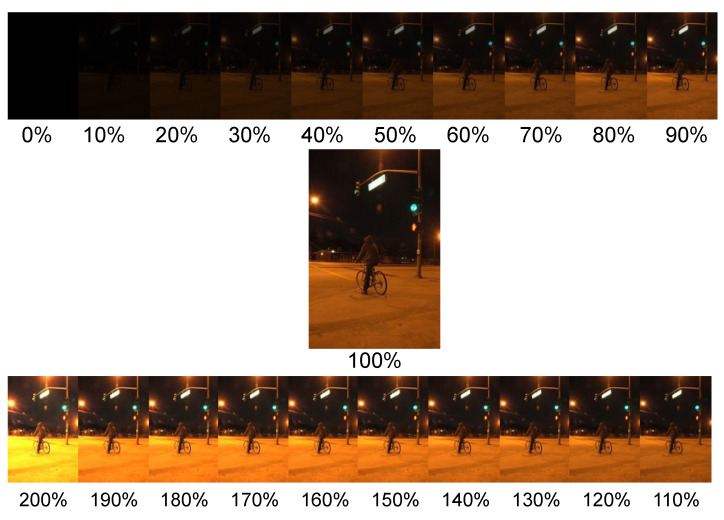
High dynamic range simulation.

In this equation, for straightforward brightness adjustment, we can set *α* to 1 and modify the value of *β*. Alternatively, you can adjust the brightness by changing the *α* value while maintaining *β* at 0, allowing you to achieve specific brightness ratios such as 200%, 190%, and so on, down to 10% or even 0% (See Figure 16).

## 4. Research Results

### 4.1. Image Processing Results

As discussed in Section 3.3, the outcomes of image enhancement, smoothing, and fusion are presented below. Similar to findings in other studies, image enhancement typically results in a lower mAP compared to the baseline. Although the human eye may perceive an improvement in image quality, nighttime image enhancement often introduces issues such as increased noise and loss of details, subsequently leading to a reduction in mAP. On the other hand, after smoothing, noise is reduced, but the overall performance still falls short of the baseline.

**Figure 17:**
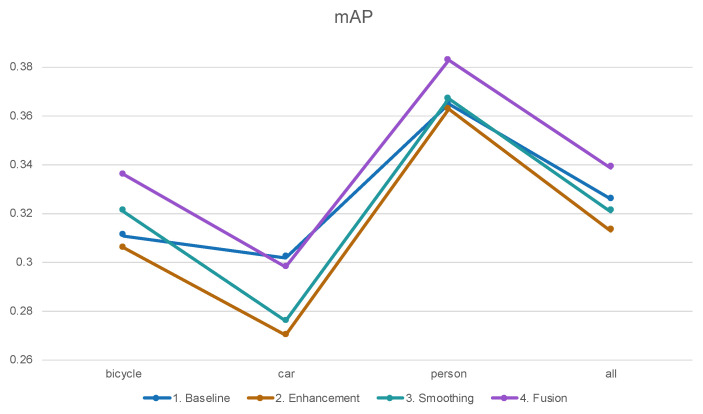
Image processing comparison on NOD dataset.

Notably, fusion outperforms smoothing, enhancement, and the baseline. This suggests that incorporating images from different datasets into deep learning training could be a promising avenue to explore (see Figure 17).

**Figure 18:**
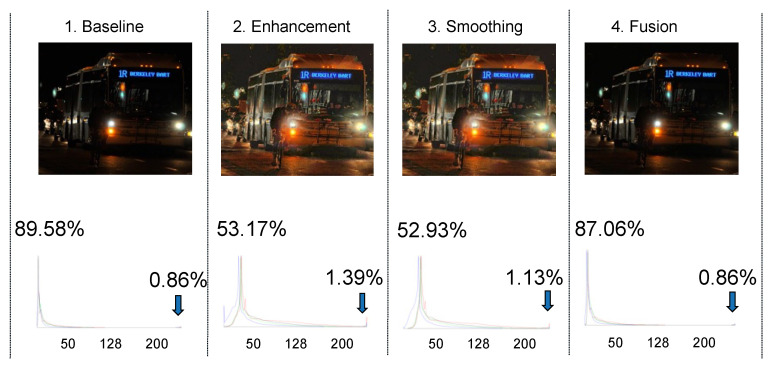
Baseline, enhancement, smoothing, and fusion images and their histograms.

Let us consider an original image depicting a night scene with a bus, bicycles, and riders in the foreground. The histogram of the original image is illustrated below. Given that it is a nighttime image, approximately 0.86% of pixel areas exhibit brightness levels exceeding 200, while a significant 89% of pixel areas have brightness levels below 50. When subjected to histogram equalization (HE), the brightness distribution shifts to the right. Although this predominantly enhances the darker regions, it also transforms medium-brightness areas into ultra-bright ones, increasing their percentage from 0.86% to 1.39%. These ultra-bright areas often lead to over-exposure noise, ultimately deteriorating the signal-to-noise ratio (S/N) (see Figure 18).

**Figure 19:**
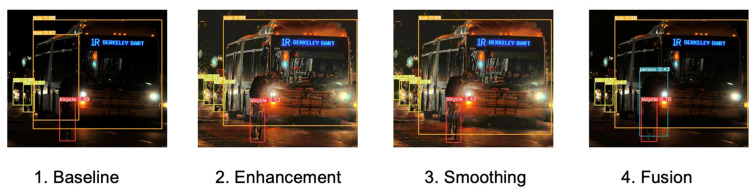
The details of detection bounding boxes.

Figure 19 shows the detection results of baseline, enhancement, smoothing, and fusion. The cyclist can be detected in fusion, which may be explained to enhance the reliability of visual signals; nocturnal insects may integrate signals over space and time at higher levels of their visual systems [6]. The enhancement and smoothing images look brighter for human eyes, but no cyclist is detected.

### 4.2. Deep Learning Model Performance Results

**Figure 20:**
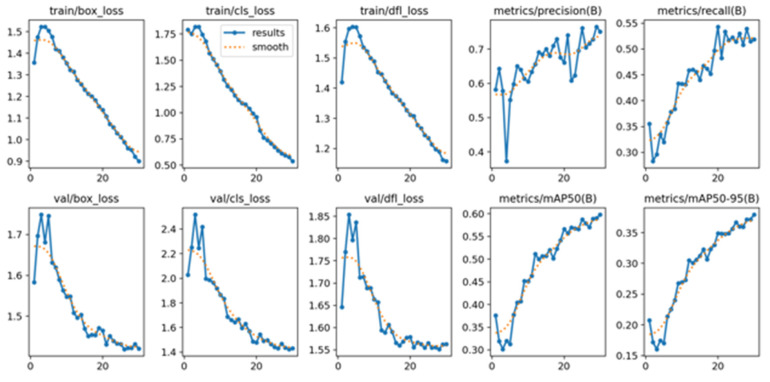
Yolo v8 training loss and precision charts.

As described in Section 3.4, this study uses Yolo v8 as the deep learning model. In the Google Colab environment, GPU acceleration is used. The loss and accuracy of the training process are shown in Figure 20.

**Figure 21:**
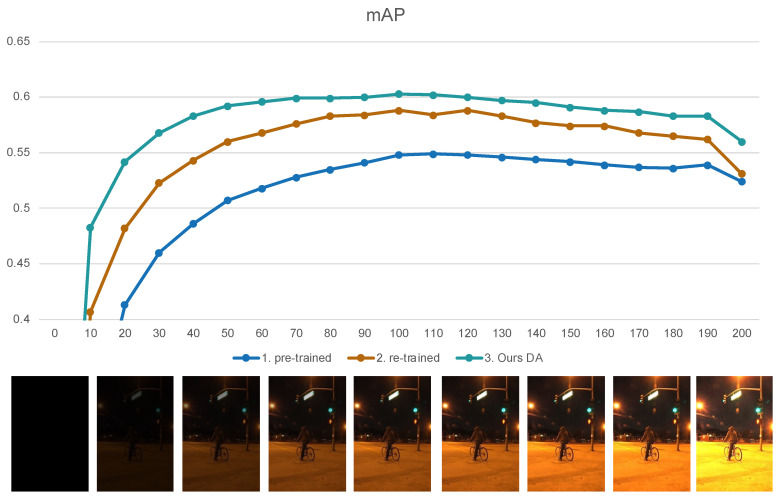
Comparison with pre-trained, re-trained, and Ours DA models on ExDark with brightness variations.

Under the test condition of original 100% illumination, the mAP values for the Yolo pre-trained model, the re-trained model by ExDark dataset, and the Ours dark adaptation model are 0.548, 0.588, and 0.603, respectively. When simulating changes in ambient brightness, as the illumination decreases, the performance of these three models also declines. At around 20−30% brightness, objects become nearly invisible to the naked eye. However, the DA model manages to maintain mAP above 0.55 under these conditions. Impressively, this model continues to achieve mAP of 0.55 even when exposed to 200% illumination, surpassing the performance of other models (see Figure 21).

To further illustrate, we randomly selected an image 2015_01524.jpg from the ExDark dataset. This photo depicts a woman sitting next to an outdoor fire pit. It appears to be taken at dusk, as the sky in the background shows the dim light after sunset.

This image, with no brightness adjustment at 100% brightness, was input into three models: pre-trained; re-trained; and DA. The results of the three images are displayed in the middle row. The pre-trained model detected a person and a bottle; the re-trained model detected a person and a cup; the DA model detected a person, a bottle, and a cup.

**Figure 22:**
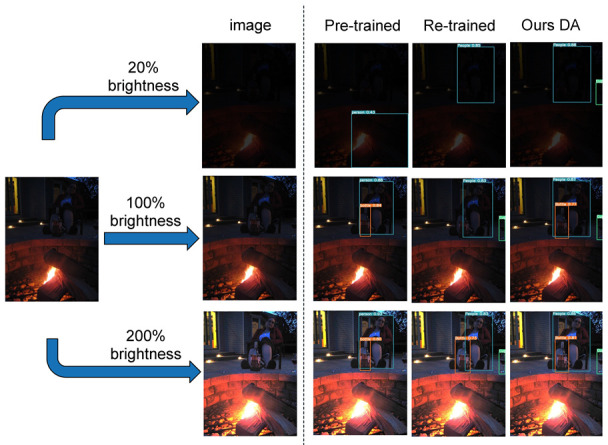
Environmental brightness simulation for detection comparison.

Due to the varying brightness of ambient light at night, as per the environmental brightness simulation in Section 3.6, one can control α and β, adjusting brightness to 20% and 200%, represented in the top and bottom rows, respectively. The pre-trained model mistakenly detected a fire pit as a person, while the re-trained model detected a person, and the DA model detected a person and a cup. In the bottom row, the pre-trained model detected a person and a bottle, and both the re-trained and DA models detected a person, a bottle, and a cup (see Figure 22).

## 5. Discussion

The innovation of this research lies in mimicking the human eye’s mechanism by overlaying the original image captured by the camera (real eye) with an image adjusted for contrast (virtual eye) to create an enhanced image for object detection. The fused image combines both original and contrast-enhanced features, effectively improving recognition rates. The traditional biological dark adaptation in vision is accomplished via the adjustment of pupils and photoreceptor cells in both eyes, taking 20 min to complete. This study using a single eye and a virtual eye combined with image processing and overlay can be completed in less than one second.

Currently, AI (artificial intelligence) has already become a trend in automation. After training data, it becomes a single model that can be used in practical applications. Regarding the learning and cognitive mechanisms of dark adaptation, since we already know the methods of image adjustment for dark adaptation, during the deep learning training, half of the images are adjusted using image enhancement to mimic the biological process of dark adaptation, thereby enabling the preemptive learning of dark adaptation adjustments.

The implications of this study extend beyond the technical sphere, addressing a real-world need for improved nighttime visibility and object detection. By simulating the rapid dark adaptation of the human eye, our approach not only enhances the capabilities of existing night-vision systems but also introduces a new paradigm for developing low-light vision technologies. This research contributes to the understanding of how artificial systems can incorporate biological principles to achieve superior performance in environments with varying light conditions. It underscores the potential for interdisciplinary approaches, combining insights from biology, artificial intelligence, and image processing to solve complex challenges.

Finally, this research addresses the significant variations in ambient brightness at night by constructing a simulated environment ranging from 0 lux to high brightness, providing a high dynamic range testing method to measure the limits of their predictive accuracy and, therefore, contributing to academic research.

## 6. Conclusions

Our study focuses on addressing the significant variations in ambient brightness during nighttime conditions. We have constructed a simulated environment ranging from 0 lux to high brightness levels, facilitating the measurement of object detection performance across a wide dynamic range. This approach offers a valuable method for high dynamic range testing, contributing to academic research.

Notably, our study does not need the generation of additional day-to-night synthetic images or the application of extra calculations for enhancing image contrast. Instead, we utilize gamma correction on half of the training data during the training process to facilitate early learning of dark adaptation and adaptability to changing lighting conditions. As a result, the dark-adapted model consistently exhibits a 1.5−6% higher mAP than the original model. This extensive range of lighting conditions allows the model to operate effectively in both twilight and night, representing a noteworthy academic advancement.

Moreover, the successful implementation of our model in simulated environments lays the groundwork for future research in real-world applications. The potential to apply our findings to actual nighttime environments, such as urban streets and rural areas, could dramatically improve safety and detection capabilities. Future work will focus on translating these bio-inspired solutions into practical tools for pedestrian protection, vehicle navigation, and broader safety applications in low-light conditions. This future direction underscores our commitment to bridging the gap between theoretical research and tangible societal benefits, particularly in enhancing nocturnal safety and visibility.

In the future, we aim to explore the potential application of various lighting models, such as diffuse, specular, and ambient lighting, to virtually illuminate specific local areas. For example, using virtual light sources to illuminate pedestrian candidate areas can significantly enhance detection accuracy. Moreover, integrating these lighting models with intelligent vehicle lighting systems can precisely illuminate targeted areas, thereby improving recognition rates. Such integration shows great promise for enhancing safety and pedestrian protection in autonomous driving scenarios, effective during both day and night.

## Data Availability

Not applicable.
